# Hyaluronic acid-modified redox-sensitive hybrid nanocomplex loading with siRNA for non-small-cell lung carcinoma therapy

**DOI:** 10.1080/10717544.2022.2032874

**Published:** 2022-02-14

**Authors:** Daoyuan Chen, Peng Zhang, Minghui Li, Congcong Li, Xiaoyan Lu, Yiying Sun, Kaoxiang Sun

**Affiliations:** a School of Pharmacy, Key Laboratory of Molecular Pharmacology and Drug Evaluation (Yantai University), Ministry of Education, Collaborative Innovation Center of Advanced Drug Delivery System and Biotech Drugs in Universities of Shandong, Yantai University, Yantai, P.R. China; b Shandong International Biotechnology Park Development Co. Ltd, Yantai, P.R. China

**Keywords:** Non-viral gene vector, siRNA, redox-sensitive nanocomplex, antitumor, safety

## Abstract

A novel hyaluronic acid (HA)-modified hybrid nanocomplex HA-SeSe-COOH/siR-93C@PAMAM, which could efficiently deliver siRNA into tumor cells via a redox-mediated intracellular disassembly, was constructed for enhanced antitumor efficacy. Thereinto, siR-93C (siRNA) and positive PAMAM were firstly mixed into the electrostatic nano-intermediate, and then diselenide bond (-SeSe-)-modified HA was coved to shield excessive positive charges. This hybrid nanocomplex displayed uniform dynamic sizes, high stability, controlled zeta potential and narrow PDI distribution. Moreover, the -SeSe- linkage displayed GSH/ROS dual responsive properties, improving intracellular trafficking of siRNA. *In vitro* assays in A549 cell line presented that HA-SeSe-COOH/siR-93C@PAMAM has low cytotoxicity, rapid lysosomal escape and significant transfection efficiency; besides, an efficient proliferation inhibition ability and enhanced apoptosis. Furthermore, in animal studies, this negative-surfaced hybrid nanocomplex showed a prolonged circulation in blood and improved inhibition of tumor growth. All these results verified our hypothesis in this study that diselenide bonds-modified HA could promote not only stability and safety of nanoparticles *in vivo* but also intracellular behavior of siRNA via redox-dual sensitive properties; furthermore, this hybrid nanocomplex provided a visible potential approach for siRNA delivery in the antitumor field.

## 1. Introduction

Recently, RNA interference (RNAi) technology has become a promising strategy for the treatment of major diseases, such as cancer, cardiovascular diseases, neurodegenerative diseases, etc. (Napoli et al., 1990; Guo et al., 2010; Kapoor et al., 2012; Kim et al., 2016; Lee et al., 2016). However, due to the existence of abundant RNase and the stability problem of RNA, including degradation during transport and transfection, RNAi-based agents are easily inactivated during systemic circulation; on the other side, the safe and effective delivery of RNAi agents, such as siRNA to target cells and efficient release into cytoplasm, still remains major hurdle for the clinical application of therapeutic RNAs, including siRNAs (Verma & Somia, 1997). Therefore, it is critical to design and develop effective RNA delivery vehicles for the clinical trials of RNAi therapeutics.

Gene delivery vectors are commonly categorized into viral and non-viral vectors (Ibraheem et al., 2014). Compared with viral vectors, non-viral vectors are less immunogenicity, easy preparation, lower cost, and higher gene encapsulation capability; all these advantages make them attractive tools for gene therapy (Kanasty et al., 2013). In the development of non-viral vectors, poly(amidoamine) (PAMAM) is an ideal candidate with high gene transfection efficiency. Because of the terminal amino groups, PAMAM molecules are positively charged at physiological pH environment, which could interact with the negatively charged phosphate groups of RNAs. Moreover, amine-terminated PAMAM also shows an ability to promote endosomal escape of loaded agents via ‘proton sponge’ effect, which could protect genes from lysosomal degradation and further enhance transfection efficiency (Shen et al., 2010). However, due to the high positive charge on the surface, the toxicity profile of PAMAM is still a major problem limiting its preclinical research and clinical applications (Lee et al., 2009; Mukherjee et al., 2010). In order to reduce the toxicity of PAMAM, surface modification and/or cation block strategies are very much required (Waite et al., 2009; Jin et al., 2011; Chang et al., 2017; Li et al., 2021). However, as the membrane potential of cells is usually negative, the decrease of the surface positive charge of vectors would lead to reduced cellular uptake and gene transfection efficiency (Jiang et al., 2009).

Stimuli-triggered dis-shielding strategies have attracted enormous interest and boosted intense studies to improve the safety of PAMAM, and redox-triggered dis-shielding is one of them, which is highly valued in cancer treatment. Compared with normal cells, most tumor cells exhibit higher levels of reactive oxygen species (ROS) and glutathione (GSH), it has been reported that the concentration of GSH in cancer cells (2–10 mM) was much higher than that in normal cells (2–20 μM) (Fang et al., 2009; Du et al., 2020; Li et al., 2021). This unique redox environment of tumor allows the breakage of redox sensitive bonds, for example, disulfide bonds or diselenide bonds, to release loaded cargoes (Shen et al., 2010; Yin et al., 2016; Li et al., 2021). More recently, diselenide bonds have emerged as attractive bio-signal sensitive linkage. Similar as disulfide bonds, diselenide bonds could be cleaved at tumor-relevant redox environment (Ma et al., 2010; Sun et al., 2017). In addition, block copolymers containing diselenide bonds also have γ-ray response capabilities, indicating that the diselenide bonds can be used to combine chemotherapy and radiotherapy (Cao et al., 2013), and it is also reported that selenium compounds can help treatment of COVID-19 (Kieliszek & Lipinski, 2020).

**Scheme 1.**
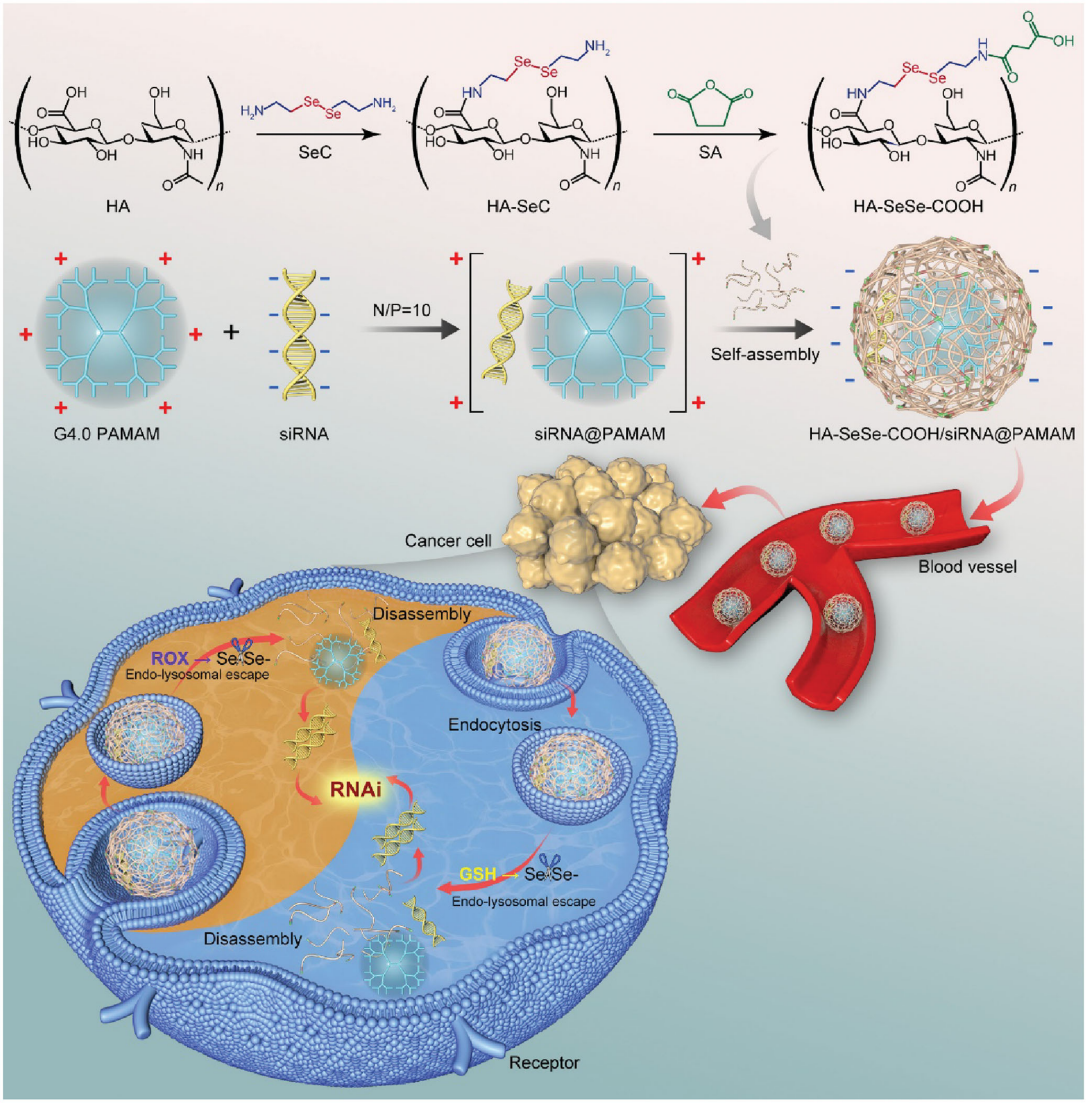
Schematic illustration of the mechanism of redox-sensitive hybrid nanocomplex loading with siRNA (HA-SeSe-COOH/siR-93C@PAMAM). HA-SeSe-COOH/siR-93C@PAMAM is constructed by siR-93C@PAMAM and HA-SeSe-COOH via electrostatic assembly process. In the redox TME, diselenide bonds-modified HA begins to break down and disassemble to re-expose the PMAMA, which could promote the lysosomal escape of loaded siRNA and to further enhance transfection efficiency.

Because of the safety problem of PAMAM associated with positive charges on the surface, we designed and synthesized a redox-sensitive anionic block copolymer to solve the biosafety limitation of PAMAM dendrimer. In the present study, PAMAM dendrimer was used to load siRNA (siR-93C@PAMAM), and the diselenide bonds-modified hyaluronic acid (HA) derivative was constructed (HA-SeSe-COOH) to conjugate with cationic siR-93C@PAMAM via electrostatic interaction (HA-SeSe-COOH/siR-93C@PAMAM) for the positive charge shielding, in order to achieve effective intracellular siRNA delivery (shown in Scheme 1). *In vitro* efficacy of the hybrid complex was investigated by monitoring particle size, stability, cytotoxicity, uptake mechanism, lysosomal escape ability, transfection efficiency and cell cycle arrest on A549 (KRAS mutation) human lung carcinoma cells. Furthermore, *in vivo* performances, such as *in vivo* distribution, tumor progression inhibition and biocompatibility, were also evaluated on A549 tumor-bearing mice.

## 2. Experimental part

### 2.1. Materials and reagents

Generation 4 PAMAM (G4 PAMAM) methanol solution (5% in methanol, w/w), RIPA Buffer, formamide, acetone, and heparin sodium were purchased from Sigma-Aldrich Co. Ltd. (St Louis, MO, U.S.A). Hyaluronic acid sodium salt (MW = 10 KDa) was purchased from Shandong Freda Biopharmaceutical Co., Ltd. (Jinan, China). N-(3-dimethylaminopropyl)-N′-ethylcarbodiimide hydrochloride (EDC·HCl), N-hydroxysuccinimide (NHS), 4-Dimethylaminopyridine (DMAP), and reduced glutathione (GSH) were purchased from Aladdin-reagent Co., Ltd. (Shanghai, China). Selenocystamine was purchased from Bidepharma Technology Co., Ltd. (Shanghai, China). 3-(4,5-dimethylthiazol2-yl)-2,5-diphenyltetrazolium bromide (MTT), InstantView™ Red Fluorescent DNA Loading Buffer, RNase A (10 mg/ml, DNase), Annexin V-FITC Apoptosis Detection Kit, Lyso-Tracker Red and phosphate buffered saline (PBS, pH 7.4) were obtained from Beyotime Biotechnology Co., Ltd. (Shanghai, China). 4% paraformaldehyde solution, 2-(4-amidinophenyl)-6-indolecarbamidine dihydrochloride (DAPI), trypsin, Chlorpromazine hydrochloride, Methyl-β-cyclodextrin, HEPES buffer (1 M), and dimethyl sulfoxide (DMSO) were obtained from Beijing Solarbio Science & Technology Co., Ltd. (Beijing, China). Dulbecco’s modified eagle medium (DMEM) cell culture, Opti-MEM Reduced Serum Media, fetal bovine serum and Lipofectamine™ 2000 was purchased from Gibco-Invitrogen (Grand Island, NY, U.S.A). Matrigel Invasion Chambers in two 24-well plates 8.0 Micron was obtained from BD BioCoat. (Franklin Lake, NJ, U.S.A). PhosSTOP phosphatase inhibitor cocktails were purchased from Roche (Basel, Switzerland). K-Ras Antibody (F234): sc-30, β-Actin Antibody (C4) Alexa Fluor® 790: sc-47778 were purchased from Santa Cruz Biotechnology (Dallas, TX, USA). Diethyl pyrocarbonate (DEPC)-treated water was obtained from Biosharp Life Sciences (Hefei, China). H_2_O_2_, luciferase-labeled A549 (A549-Luc) cells and A-549 (KRAS mutation) were kindly provided by Luye life group. (Yantai, China). RNase-free diethyl pyrocarbonate (DEPC)-treated water was used for all solutions containing siRNA and dendrimers.

The siR-93C (KRAS siRNA) consisted of the following sequences: KRAS sense 5′-CAGCUAAUUCAGAAUCAUU-3′, and antisense 5′-AAUGAUUCUGAAUUAGCUG-3′ and the negative control siRNA sequences: sense 5′-UUCUCCGAACGUGUCACGUTT-3′, and antisense 5′-ACGUGACACGUUCGGAGAATT-3′ were purchased from Shanghai GenePharma Co., Ltd. (Shanghai, China).

### 2.2. Synthesis of HA-Se-Se-COOH block copolymer

EDC (15.5 mg), NHS (11.5 mg), and DMAP (0.6 mg) were added to HA (50.9 mg) in 10 ml formamide with stirring for 2 h to dissolve completely at room temperature. Selenocystamine (24.6 mg) was added and the whole mixture was stirred for 12 h, then the reaction mixture was concentrated under reduced pressure and was poured into acetone to precipitation. Next, the dried white precipitate (49.7 mg), succinic anhydride (12.4 mg), and triethylamine (12.6 mg) were dissolved completely in 10 ml formamide, then the mixture was stirred at 60 °C for 3 h. After that, the mixture was precipitated in acetone to obtain the final product HA-SeSe-COOH (He et al., 2014; Sun et al., 2019). The chemical structure of HA-SeSe-COOH was determined by hydrogen nuclear magnetic resonance (^1^H-NMR) spectroscopy and Fourier transform infrared (FT-IR) spectroscopy.

### 2.3. Assembly of HA-SeSe-COOH/siR-93C@PAMAM complexes

siRNA (siR-93C) was diluted in RNase/DNase-free sterile microcentrifuge tubes to provide a final concentration of 0.05 mg/mL. The complexation of siR-93C with G4 PAMAM was undertaken at the N/P ratio of 0.5,1,2,4,6,8,10 in 10 mM HEPES buffer, pH 7.4 as previously reported (Jensen et al., 2010; Pavan et al., 2010; Jensen et al., 2011). Briefly, a total volume of 500 μL siR-93C solutions was gently added to the PAMAM solution, followed by 20 s of vortex mixing and further incubated at room temperature for 20 min to form siR-93C@PAMAM. HA-SeSe-COOH then was gently added into the pre-prepared siR-93C@PAMAM to construct HA-SeSe-COOH/siR-93C@PAMAM complexes by electrostatic self-assembly. For example, specific weight of HA-Se-Se-COOH was added to siR-93C@PAMAM solution, vortexed for 30 seconds, and incubated for 20 more minutes at room temperature for complete HA-SeSe-COOH/siR-93C@PAMAM complex formation. HA-SeSe-COOH/siR-93C@PAMAM complexes with various ratios of HA-SeSe-COOH to siR-93C@PAMAM (w/w) from 0.2 to 4 were achieved in this study.

### 2.4. Physicochemical properties evaluation of HA-SeSe-COOH/siR-93C@PAMAM

#### 2.4.1. Size and zeta potential measurements

The particle size and zeta potential of siR-93C@PAMAM and HA-SeSe-COOH/siR-93C@PAMAM were measured by Malvern Instruments (Zetasizer Nano ZS, Malvern, UK). Complexes aggregation was determined by the polydispersity index (PDI) value. The morphology of HA-SeSe-COOH/siR-93C@PAMAM was characterized by transmission electron microscopy (TEM, JEM-1400 Plus, Tokyo, Japan).

#### 2.4.2. Gel electrophoresis assay

The siRNA compaction capability of PAMAM was evaluated by agarose gel retardation assay. siR-93C@PAMAM complexes were prepared with the final siRNA concentration of 0.05 mg/mL. Different N/P complexes solutions (10 μL) were analyzed by 1.5% agarose gel electrophoresis (100 V, 30 min) (Ziraksaz et al., 2013). Then, the gel was stained with Fluorescent DNA loading buffer, and the stained bands were visualized under ultraviolet (UV) illumination light.

#### 2.4.3. Redox sensitivity of HA-SeSe-COOH/siR-93C@PAMAM

HA-SeSe-COOH/siR-93C@PAMAM were treated with L-Glutathione (GSH concentration 20 μM or 10 mM) (Huang et al., 2018; Du et al., 2020; Zhang et al., 2020; Li et al., 2021), H_2_O_2_ (7.0 μmol/L or 1.0 mmol/L) (Mueller, 2000; Boveris et al., 2002; Martin & Barrett, 2002; Stone & Yang, 2006; Giorgio et al., 2007), and normal saline, respectively. The particle size and the polydispersity index of five groups were measured every 30 min to evaluate the redox sensitivity of HA-SeSe-COOH/siR-93C@PAMAM.

### 2.5. *In vitro* evaluation of HA-SeSe-COOH/siR-93C@PAMAM

#### 2.5.1. Cell culture and viability assay

In this study, cell viability was determined by MTT assay (Bansal et al., 2014). A549 cell line was cultured in DMEM medium containing 10% fetal bovine serum (FBS) at 37 °C with 5% CO_2_ in a humidified incubator. In the cell viability assay, A549 cells were seeded at 0.35 × 10^4^ cells/well in 96-well plates and cultured overnight. When cells were fully adhered, the medium was replaced with fresh medium, and siNC@PAMAM or HA-SeSe-COOH/siNC@PAMAM were added with the final siNC concentration of 10, 50, 100, 150, 200 nM for an additional 48 h. Subsequently, 20 μL of MTT solution (5 mg/mL) was added to all wells, and after 4 h incubation, the supernatant was removed, and the culture medium was replaced by 150 μL DMSO to dissolve the formazan crystal. Finally, the UV absorbance was measured at 570 nm using the microplate reader (Model 550, Bio-Rad, U.S.A), and the untreated group was chosen as control group to calculate the cell viability.

#### 2.5.2. *In vitro* cellular uptake mechanism assay

To investigate the cellular uptake mechanism of HA-SeSe-COOH/siR-93C@PAMAM, different inhibitors such as chlorpromazine (CPZ), methyl-β-cyclodextrin (M-β-CD), and 5-N-ethyl-N-isoproamiloride (EIPA) to block clathrin, caveolin, and macropinocytosis-mediated endocytosis pathway, respectively. A549 cells were seeded in 6-well plates at 2 × 10^5^ cells/well and cultured for 24 h. Then cell culture medium was replaced with serum-free medium containing different endocytic inhibitors, CPZ (0.035 mM), M-β-CD (3 mM), and EIPA (0.25 mM). After 30 min pretreatment, HA-SeSe-COOH/siR-93C@PAMAM with the same amount of siRNA concentration (100 nM) were exposed to cells and incubated for additional 4 h at 37 °C. A549 cells were also preincubated with HA (0.1 mM) for 1 h to evaluate the influence of CD44 receptor on cellular uptake of HA-SeSe-COOH/siR-93C@PAMAM (Li et al., 2021). Subsequently, the cells were washed three times with PBS, harvested with trypsin and analyzed by flow cytometry to detect the MFI of siRNA, which was labeled by FAM.

#### 2.5.3. Lysosomal escape

A549 cells were plated at 2 × 10^5^ cells/well in a 6-well plate and cultured overnight. Then, the medium was replaced with 2 mL of fresh serum-free medium containing complexes. The complexes containing medium were discarded after certain period incubation and cells were washed with PBS twice. Next, cells were incubated with LysoTracker Red for 1 h at 37 °C to stain lysosome. Subsequently, cells were fixed with 4% paraformaldehyde and nuclei were stained with DAPI for 30 min before being observed under Confocal Laser Scanning Confocal Microscopy (CLSM) (Shi et al., 2018), and siR-93C was labeled by FAM. Commercial transfectionreagent Lipofectamine-2000 (Lipo2000) was used as the parallel control group.

#### 2.5.4. *In vitro* siRNA transfection assay

A549-Luc cells were seeded into 96-well plates at a density of 0.3 × 10^4^ cells per well in 200 μL complete culture medium. After 24 h, culture medium was removed, and the cells were cleaned with PBS twice. Then, 100 μL of fresh complexes was added to each well (final siRNA concentration was 100 nM). 4 h later, the transfection medium was replaced with fresh complete DMEM medium. After 48 h incubation, cells were washed with PBS twice and lysed with luciferase cell culture lysis buffer (He et al., 2015). After 10 min incubation without light later, the luciferase activity was decided by microplate reader (Model 550, Bio-Rad, USA).

#### 2.5.5. Wound healing assay

A549 cells were seeded in 6-well plates at a density of 2 × 10^5^ cells/well and cultured 24 h to reach 90% confluence as a monolayer. Then the monolayer was scratched with a sterile pipette tip (200 μL type) and washed with PBS twice (Wang et al., 2015; Li et al., 2020). The cells were treated by the complexes and continued to culture in DMEM containing 10% FBS for 72 h and the untreated group was used as Control group, the wound areas were measured by microplate reader (Model 550, Bio-Rad, U.S.A) at 12, 24, 36, 48, 60 and 72 h, respectively.

#### 2.5.6. Transwell migration assay

The cell culture and transfection process were performed as described previously. After trypsinization, A549 cells were plated in the upper chamber inserted in 24-well plates with serum-free DMEM at a density of 2.0 × 10^4^ cells/well, and DMEM with 10% FBS was added into the lower chamber. After incubation for 24 h, cells in the upper chamber were wiped by cotton swab, and cells migrated to the lower surface were fixed with ethanol at 4 °C for 15 min and stained with 0.2% crystal violet for 30 min (Wang et al., 2015; Li et al., 2020). The stained cells were then rinsed with PBS and recorded by Olympus fluorescence microscope. The stained cells were dissolved in 33% (v/v) acetic acid solution and measured on microplate reader at 578 nm, and untreated group was used as the Control group.

#### 2.5.7. Cell cycle analysis

A549 cells (2 × 10^5^ cells/well) were exposed to various complexes solution (final concentration of siR-93C was 100 nM) for 24 h. Then, the harvested cells were washed with PBS and fixed with 70% ethanol at −20 °C for 1 hour. The fixed cells were centrifuged and washed with PBS three times. One unit of RNase was added to cell suspension and incubated for 30 min at 37 °C, then the cells were stained with PI solution (200 μL) for 30 min in the dark. Subsequently, a flow cytometer was used to analyze the cell cycle distribution.

#### 2.5.8. Apoptosis assay

A549 cells were seeded in 6-well plates at a density of 2.0 × 10^5^ cells/well and cultured overnight. Then, 2 ml of different complexes which was diluted in serum-free DMEM was added to each well for 6 h incubation. After trypsinization, the medium was removed, and cells were washed with PBS twice. Then, cells were resuspended in binding buffer and stained by FITC/PI staining solution at room temperature for 15 minutes without light. The cells were analyzed by flow cytometry within 1 hour to detect cell apoptosis ratio.

#### 2.5.9. Western blotting

A549 cells were cultured and transfected as described in section 2.5.2. The transfected cells were rinsed with PBS and the lyzed with RIPA lysis buffer containing protease and phosphatase inhibitor for 30 min, and the protein concentration was quantified by bicinchoninic acid (BCA) detection kit. Equal amounts of total protein were subjected to separate by electrophoresis on the polyacrylamide gels and transferred to PVDF membrane. After blocking with 5% skim milk dissolved in TBST (TBS buffer containing 0.1% Tween-20) for 1 h, the membrane was incubated overnight at 4 °C with the corresponding primary antibodies, and secondary antibodies were incubated for 1 h at room temperature. The expression level of related proteins was detected using the enhanced chemiluminescence kit on a Tanon-5200 Image Analyzer, the protein bands were analyzed using ImageJ software (Mehta et al., 2019).

### 2.6. *In vivo* antitumor efficacy study

#### 2.6.1. Animal model

BALB/c nude mice (male, 18.0–20.0 g, 5–6 weeks) were used in *in vivo* study. All animal experiments and research procedures are in accordance with the corresponding national standards. The A549 tumor-bearing nude mice model was established by subcutaneously injection of A549 cells (5.0 × 10^6^ per mouse) into the right armpit region (photographs of tumor-bearing mice were shown in Supporting Information). The tumor volume (*V*) was calculated by the following equation:

$$V=\frac{\mathrm{length}\times {\mathrm{width}}^{2}}{2}$$

#### 2.6.2. Biodistribution

The *in vivo* biodistribution studies were performed when tumor volumes reached approximately 100 mm^3^. *In vivo* distribution of complexes was determined by fluorescent image of siRNA labeled by FAM (siR-93C-FAM). A549 tumor-bearing nude mice were injected via tail vein with siR-93C-FAM@Lipo2000, siR-93C-FAM@PAMAM, and HA-SeSe-COOH/siR-93C-FAM@PAMAM (siRNA concentration was 100 nM, 100 μL). After tail vein injection, the *in vivo* fluorescence images were taken at certain predetermined time. At 24 h, the tumor tissues and major organs were harvested and imaged for the detailed distribution study.

#### 2.6.3. *In vivo* antitumor effect and biosafety study

When the xenografted tumors grew to about 100 mm^3^, the nude mice were randomly divided into four groups (*n* = 5) which were treated with saline, siR-93C@Lipo2000, siR-93C@PAMAM and HA-SeSe-COOH/siR-93C@PAMAM via tail intravenously injection (final siRNA: 100 nM, 100 μL) every other day until 21 days. The therapeutic effect of each group was monitored by measuring the tumor volumes. Moreover, the body weight of the A549 tumor-bearing nude mice was also collected to assess the systemic toxicity of virous complexes. The mice were sacrificed on day 21, tumors and major organs were excised, fixed in 4% paraformaldehyde and paraffin embedded. The histological slices were stained with hematoxylin and eosin (H&E), and then to investigate the antitumor effect and *in vivo* safety of different siRNA formulations.

### 2.7. Statistical analysis

Data were presented as mean ± standard deviations (SD). Statistical comparisons between two groups were evaluated using unpaired Student's t-test (two tailed) and performed by GraphPad Prism 8.0. A value of *p* < 0.05 was considered statistically significant.

## 3. Results and discussion

### 3.1. Synthesis of HA-SeSe-COOH

**Figure 1.**
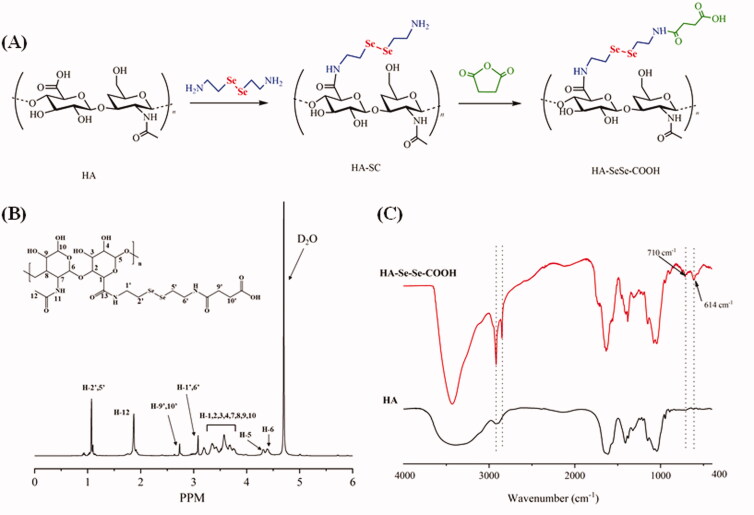
Synthesis and characterization of HA-SeSe-COOH: (A) Synthetic route of HA-SeSe-COOH; (B) ^1^H-NMR spectrum, and (C) FTIR spectrum of HA-SeSe-COOH.

HA-SeSe-COOH was synthesized following the route in Figure 1(A), and the chemical structure of the product was determined by ^1^H-NMR and FT-IR spectroscopy. Figure 1(B) shows ^1^H-NMR spectrum of HA-SeSe-COOH. From ^1^H-NMR spectrum, characteristic peaks from 3.16 − 3.75 ppm were attributed to the hydrogen (H−1,2,3,4,7,8,9,10) from D-glucuronic acid unit of HA, resonance at 4.39 ppm and 4.31 ppm were from H-6 and H-5 of the hyaluronic acid. Signal at 3.08 ppm was assigned to H-1′ and H-6′ which were connected to secondary amine, methyl group (H-12) could be seen at 2.01 ppm. In addition, resonance at 1.07 ppm was corresponded to the protons of H-2′ and H-5′ which were attached to -*SeSe*-. ^1^H-NMR result confirmed the successful synthesis of HA-SeSe-COOH. In the FT-IR spectra of HA and HA-SeSe-COOH (Figure 1(C)), the broadband in the region of 3000 − 3700 cm^−1^ was attributed to the stretching vibration of the hydroxyl groups of HA, peaks at 2859 cm^−1^ and 2916 cm^−1^ were from the stretch vibration of -*CH_2_*- groups from Selenocystamine. The characteristic absorption peak of -*Se*-*Se*- at 710 cm^−1^ and stretch vibration of -*CH_2_*-*Se*- at 614 cm^−1^ indicated the successful construction of HA-SeSe-COOH.

### 3.2. Characterization of HA-SeSe-COOH/siR-93C@PAMAM hybrid nanocomplex

**Figure 2.**
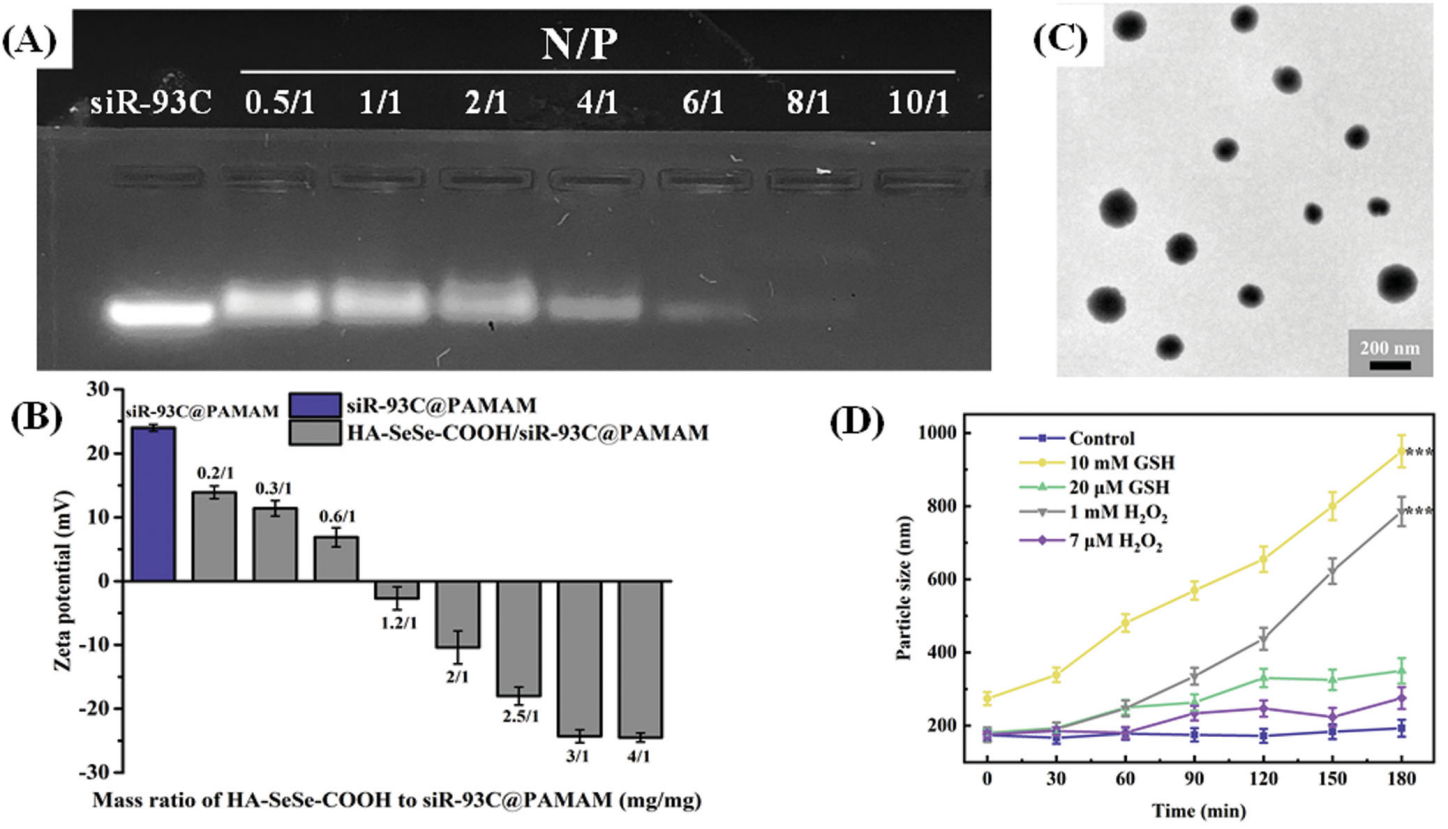
(A) Gel electrophoresis assay of siR-93C@PAMAM nanocomplex at different N/P ratios. (B) Zeta potentials of HA-SeSe-COOH/siR-93C@PAMAM with different mass ratios of HA-SeSe-COOH to siR-93C@PAMAM. (C) TEM image of HA-SeSe-COOH/siR-93C@PAMAM (with the ratio of 3/1). (D) Particle size of HA-SeSe-COOH/siR-93C@PAMAM under different GSH and ROS conditions.

To design a complex vector taking advantage of PAMAM, siR-93C@PAMAM complexes were constructed at various nitrogen to phosphorus ratios (N/P, the mole ratio of the amine groups of PAMAM to the phosphate groups of siRNAs) to determine the optimum amount of siRNA and PAMAM. The optimized N/P was determined by agarose gel electrophoresis (in Figure 2(A)). The results show that when the N/P ratio was greater than 10, no free siRNA signal could be detected by agarose gel electrophoresis, indicating that siRNA was fully loaded. Thus, in this work the ideal N/P ratio was 10.

Under this N/P ration, the particle size of constructed siR-93C@PAMAM complex was 149 nm (PDI = 0.114), and the zeta potential of complex was +24.7 mV (shown in Figure 2(B)). Further, in order to reduce the surface positive charge to improve the stability of siRNA during blood circulation, HA-SeSe-COOH was introduced as the outer shell to cover the inner core siR-93C@PAMAM; at the same time, the particle size of HA-SeSe-COOH/siR-93C@PAMAM would be increased also. To optimize the employed amount of HA-SeSe-COOH, different mass ratios of HA-SeSe-COOH to siR-93C@PAMAM were tested between 0.2/1 and 4/1. The particle size results are shown in Table 1. It was found that when the ratio was from 0.6/1 to 1.2/1, there was no nano association complex constructed in the system, this may be caused by the random aggregation of siR-93C@PAMAM and HA-SeSe-COOH when the zeta potential of mixture close to zero. On the other side, the amount of HA-SeSe-COOH could not only affect the stability of complex, but also affect the surface charge and particle size of HA-SeSe-COOH/siR-93C@PAMAM. It was found that when the HA-SeSe-COOH was less than siR-93C@PAMAM, the zeta potential of complex was positively charged even the particle size could be similar to each other. For example, when the mass ratio of HA-SeSe-COOH to siR-93C@PAMAM was 0.3/1 and 3/1, complexes with similar particle size (197 nm and 181 nm, respectively) would be obtained, however, the zeta potential was markedly different, positive complex (+12.4 mv) was constructed when the ratio was 0.3/1 compared with negatively charged complex (–24.6 mv) with the ratio of 3/1. Considering the issue of biological safety (which will be discussed in the next section), the ratio of 3/1 was used in the following study, which the siRNA-loading efficiency was 3.23%. The morphology of HA-SeSe-COOH/siR-93C@PAMAM (with the ratio of 3/1) was observed by TEM to further obtain the nanostructure information. TEM image (Figure 2(C)) showed that HA-SeSe-COOH/siR-93C@PAMAM was spherical particle with the obvious nano structure.

**Table 1.** Particle size and PDI of HA-SeSe-COOH/siR-93C@PAMAM with different mass ratios of HA-SeSe-COOH to siR-93C@PAMAM.

	siR-93C@PAMAM	HA-SeSe-COOH/siR-93C@PAMAM							
		0.2/1	0.3/1	0.6/1	1.2/1	2.0/1	2.5/1	3.0/1	4.0/1
Particle size (nm)	149 ± 3.5	224 ± 5.7	197 ± 4.1	884 ± 56.0	3394 ± 602.0	511.9 ± 7.2	331 ± 5.8	181 ± 3.8	244.8 ± 7.4
PDI	0.11	0.05	0.12	2.72	15.23	0.86	0.57	0.19	0.09

### 3.3. Redox sensitivity of HA-SeSe-COOH/siR-93C@PAMAM

Intracellular disassemble behavior of this hybrid nanocomplex is considered as a key step for siRNA delivery. Redox-stimuli cleavage could have the function enhancing siRNA release into cytoplasm, upgrading final transfection. Figure 2(D)) shows the redox sensitivity of HA-SeSe-COOH/siR-93C@PAMAM under different GSH or ROS conditions with 180 min. In the PBS buffer (Control group), there was no observed change in the size until 180 minutes. However, the particle size of HA-SeSe-COOH/siR-93C@PAMAM began to increase slowly under low concentration of GSH or ROS, which was due to the redox-responsive cleavage of diselenide bonds. When the GSH or ROS level was improved, the particle size increased dramatically, indicating the dissociation of HA-SeSe-COOH/siR-93C@PAMAM. Moreover, given the intracellular ROS and GSH level of cancer cells, the responsive dissociation ability of HA-SeSe-COOH/siR-93C@PAMAM could enable protection and stability during blood circulation and efficient release of siR-93C@PAMAM at the tumor site.

### 3.4. *In vitro* cytotoxicity

**Figure 3.**
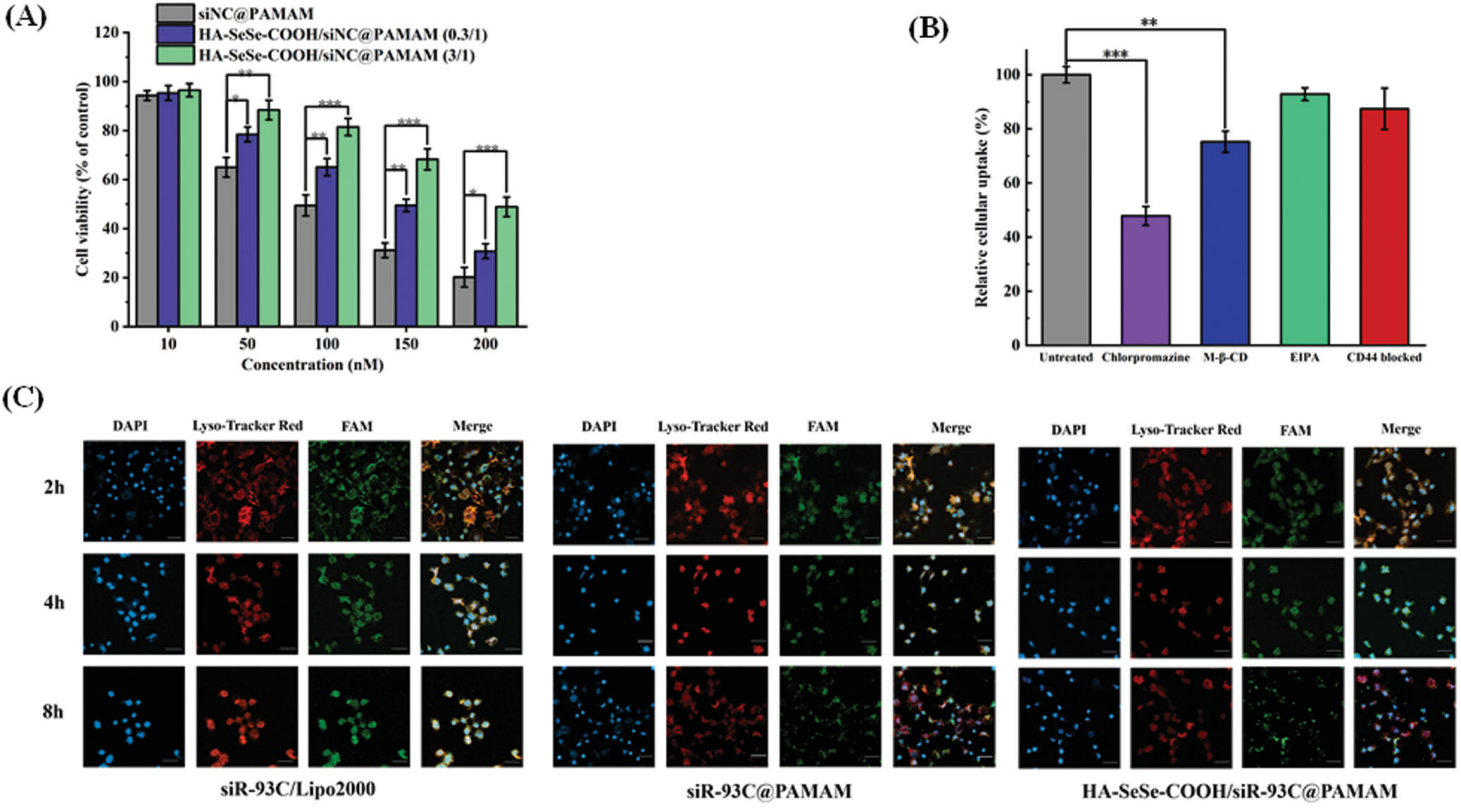
(A) Cytotoxicity of various concentrations of siNC@PAMAM and HA-SeSe-COOH/siNC@PAMAM in A549 cells from 10 to 200 nM. (B) Effect of different endocytosis inhibitors on the cellular uptake of HA-SeSe-COOH/siR-93C@PAMAM. (C) Lysosomal escape ability of siR-93C@Lipo2000, siR-93C@PAMAM and HA-SeSe-COOH/siR-93C@PAMAM against A549 cells, the blue fluorescence signal represents cell nucleus, red fluorescence represents the stained lysosome, and green fluorescence signal represents siR-93C which was labeled by FAM (*p < 0.05, **p < 0.01, ***p < 0.001, scale bar: 100 μm).

To investigate the *in vitro* cytotoxicity of different nanocomplexes, siNC was used to avoid the interference of siR-93C. *In vitro* cytotoxicity of various concentrations of siNC@PAMAM and HA-SeSe-COOH/siNC@PAMAM was evaluated by MTT assay in A549 cells. From Figure 3(A) it could be found that the cytotoxicity of both siNC@PAMAM and HA-SeSe-COOH/siNC@PAMAM was concentration dependent. Compared with HA-SeSe-COOH/siNC@PAMAM, the cytotoxicity of siNC@PAMAM is generally higher at the same siNC concentration, which was due to electrostatic attraction between negatively charged cell membrane and positively charged siNC@PAMAM under physiological conditions as the extensive number of amino groups on the surface of PAMAM. As expected, the cytotoxicity of HA-SeSe-COOH/siNC@PAMAM was significantly reduced by increasing amount of HA-SeSe-COOH, indicated that the shell of HA-SeSe-COOH would considerably enhance the biocompatibility of PAMAM. When the siNC was 100 nM, the survival of A549 cells was still 81.5% in HA-SeSe-COOH/siNC@PAMAM (3/1) group, which was obviously higher than that of 65.2% in HA-SeSe-COOH/siNC@PAMAM (0.3/1) group and 49.5% (siNC@PAMAM). Therefore, HA-SeSe-COOH/siNC@PAMAM (3/1) was selected in the following study. The *in vitro* cytotoxicity results suggested that PAMAM cytotoxicity can be reduced by the HA-derivate shell.

### 3.5. Cellular uptake pathway investigation

The cellular uptake pathway of nanocomplex plays a critical role in the final transfection efficacy. In this study, several endocytosis inhibitors were used to investigate the endocytosis pathway of HA-SeSe-COOH/siR-93C@PAMAM, and FAM-labeled siR-93C was introduced for the internalization investigation. As shown in Figure 3(B), Chlorpromazine treatment showed significant effect in the cellular uptake of complex, with the cellular uptake of siR-93C loaded complex was reduced to 47.86%, which was much lower than that of M-β-CD treated group (cellular uptake was still 77.23%), indicating that HA-SeSe-COOH/siR-93C@PAMAM was mainly internalized via clathrin-mediated endocytosis pathway. This cellular uptake mechanism results were similar to our previous study (Li et al., 2021). Moreover, less HA-SeSe-COOH/siR-93C@PAMAM was detected when the CD44 receptor was blocked by pretreatment with free HA, showing that HA-CD44 binding would improve the internalization of HA-SeSe-COOH/siR-93C@PAMAM nanocomplex.

### 3.6. Lysosomal escape

It is well known that the internalized RNA and/or drugs are easily degraded by lysosome enzymes, such as acid RNases and exonucleases, resulting in reduced anti-cancer efficacy (Huang et al., 2020). Thus, lysosomal escape ability of siRNA loaded complex is crucial for enhancing the therapeutic efficacy. The lysosomal escape ability study was investigated using confocal laser scanning microscope (CLSM). From the CLSM images (shown in Figure 3(C)), the yellow signal, overlap area of red fluorescence (lysosome) and green fluorescence (green fluorescent FAM labeled siR-93C), indicated that siR-93C was trapped in lysosome; after siR-93C escaped from lysosome, bright cyan fluorescence could be observed from CLSM image, also the separation of green fluorescence with red region was detected (shown in Figure S1). In siR-93C@Lipo2000 group, siR-93C was mainly present in the lysosome at 2 h. Although there was slight cyan fluorescence signal could be observed at 4 h, the stronger yellow signal indicated that most of the siRNA was still localized in lysosome until 8 h. In the siR-93C@PAMAM and HA-SeSe-COOH/siR-93C@PAMAM groups, a fast and effective lysosomal escape could be observed at even 2 h as the cyan signal was clearly observed from CLSM indicating the spread out of the siR-93C from lysosome. The effective lysosomal escape ability was accounted by the proton sponge effect” of PAMAM. Though the HA-SeSe-COOH shell may affect the efficiency of lysosome escape, the GSH-responsive disassembly of the shell and re-exposure of siR-93C@PAMAM in the tumor intracellular environment could still trigger the lysosome escape.

### 3.7. Transfection behavior of HA-SeSe-COOH/siR-93C@PAMAM

**Figure 4.**
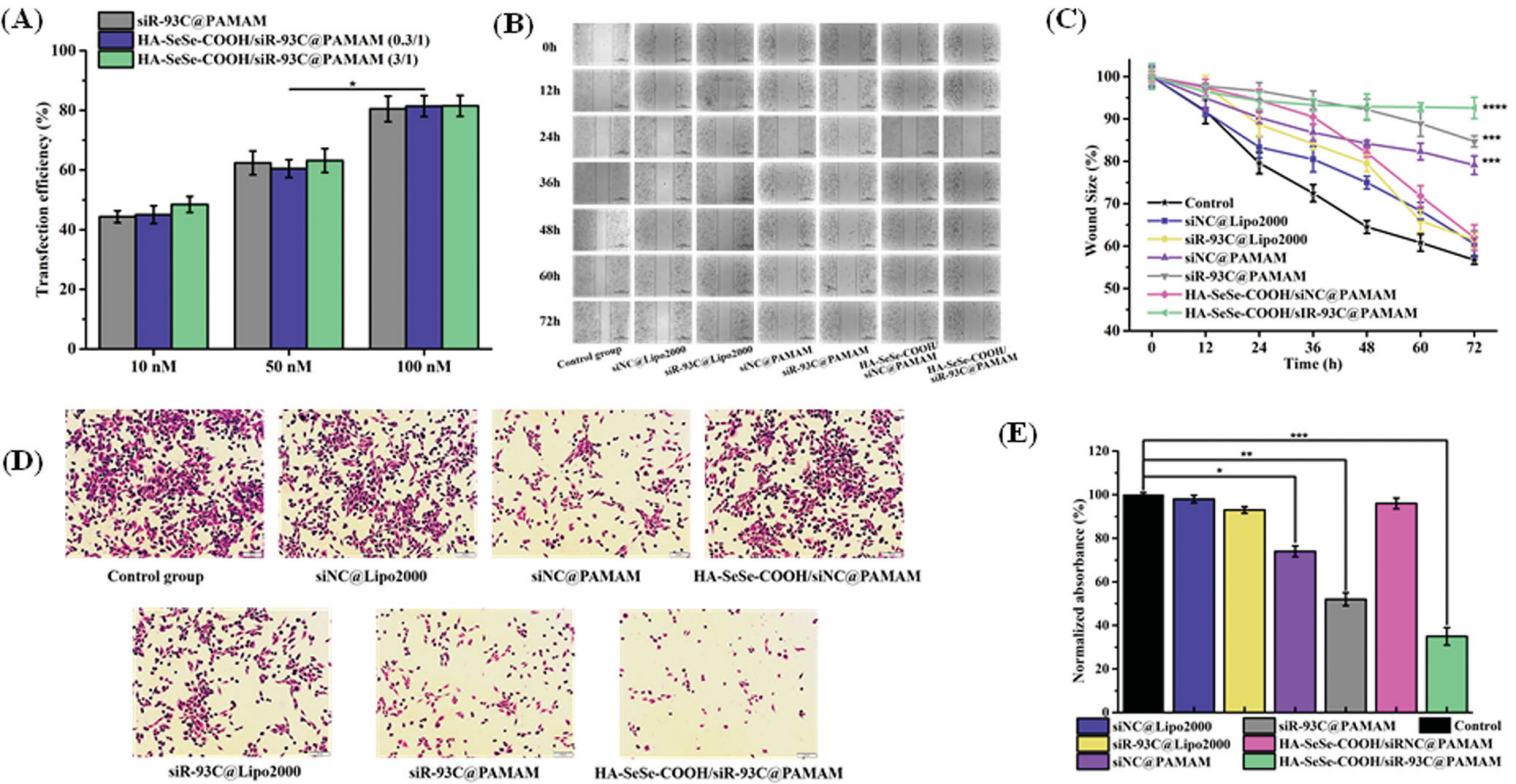
(A) Transfection efficiency of different concentrations of siR-93C@PAMAM and HA-SeSe-COOH/siR-93C@PAMAM from 10 to 100 nM. (B) Wound healing assay. Scale bar: 200 μm. (C) Wound scratch change of different complex (***p < 0.001, ****p < 0.0001). (D) Transwell migration assay. Scale bar: 20 μm. (E) Transwell migration analysis (*p < 0.05, **p < 0.01, ***p < 0.001).

Luciferase activity assay was performed to investigate the transfection efficiency of HA-SeSe-COOH/siR-93C@PAMAM. As shown in Figure 4(A), the results showed that the transfection efficiency was improved when increasing the siRNA concentration, all the group showed the highest transfection efficiency when the siR-93C concentration was improved to 100 nM, which was almost 2-fold as compared to 10 nM. Surprisingly, luciferase expression in siR-93C@PAMAM, HA-SeSe-COOH/siR-93C@PAMAM (0.3/1) and HA-SeSe-COOH/siR-93C@PAMAM (3/1) was no significant difference. It has been found that the positively charged particles strongly interact with cells and penetrate into the cells more effectively than negatively charged particles. In this study, siR-93C@PAMAM and HA-SeSe-COOH/siR-93C@PAMAM (0.3/1) were positively charged, whereas the HA-SeSe-COOH/siR-93C@PAMAM (3/1) was obviously negatively charged. The possible explanation for transfection result was that the penetration ability of HA-SeSe-COOH/siR-93C@PAMAM (3/1) into A549 cells could be improved by the hyaluronic acid – derivative shell, as hyaluronic acid could specifically bind to CD44 which was overexpressed on A549 cells surface (Cano et al., 2020).

### 3.8. Cell migration and invasion *in vitro*

Compared to other tumor cells, strong invasiveness and high migration ability were observed in A549 cells. Thus, the inhibitory capacity of migration and invasion of A549 cells by HA-SeSe-COOH/siR-93C@PAMAM were evaluated.

#### 3.8.1. Wound healing assay

In order to study the migration ability of A549 cells transfected by siR-93C/Lipo2000, wound scratches were observed and measured every 12 h after transfection. As shown in Figure 4(B,C), the cells in Control group displayed the strongest migration ability, and the siNC@Lipo2000 also exhibited obvious healing trend, whereas the siNC@PAMAM, siR-93C@PAMAM and HA-SeSe-COOH/siR-93C@PAMAM groups showed obvious inhibitory effects of migration. At 72 h, the wound scratch ratio of the control group was 56.7%, whereas the wound scratch ratios of the siR-93C@PAMAM and HA-SeSe-COOH/siR-93C@PAMAM groups were still 84.7% and 92.6%, respectively. The relative larger wound size in HA-SeSe-COOH/siR-93C@PAMAM group indicated the stronger ability of inhibiting A549 cells migration, which was due to the successful delivery and effective expression of siRNA in the cells.

#### 3.8.2. Transwell assay

Transwell migration assay was also performed to further investigate the anti-invasive effects in A549 cells after the siR-93C transfection (Figure 4(D,E)). It can be seen that the cells still exhibited strong invasive ability in the Control group, siNC group and HA-SeSe-COOH/siNC@PAMAM group. Nevertheless, the number of cells passing through the chamber in the siNC@PAMAM, siR-93C@PAMAM and HA-SeSe-COOH/siR-93C@PAMAM groups were dramatically decreased, with ratios were only 74.5%, 52.7%, and 35.2%, respectively. It should be noted that the number of penetrated cells in the siNC@PAMAM group was relatively lower than other groups, which was related to the cytotoxicity of PAMAM itself. The Transwell cell invasion results showed that siRNA transfection via HA-SeSe-COOH/siR-93C@PAMAM delivery could decrease the invasion ability of A549 cells.

### 3.9. Cycle arrest and apoptosis

**Figure 5.**
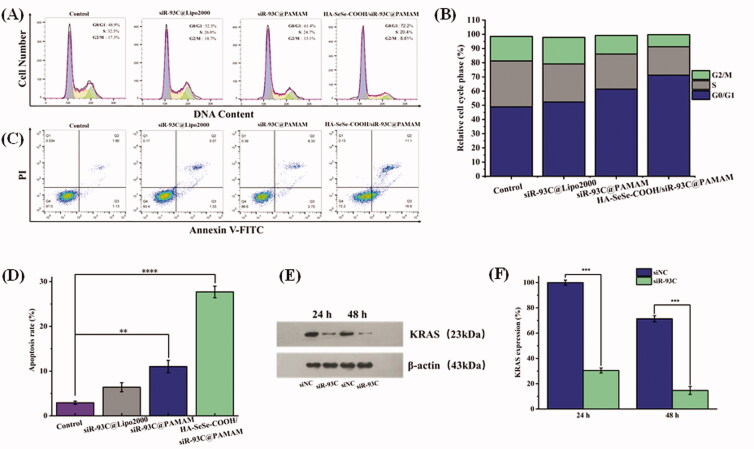
(A) Histograms of cell cycle distribution after the treatment with control, siR-93C@Lipo2000, siR-93C@PAMAM, and HA-SeSe-COOH/siR-93C@PAMAM. (B) Relative distribution of cell population in the cell cycle phase. (C) Cell apoptosis and (D) Cell apoptosis rate of A549 cells after the treatment with control, siR-93C@Lipo2000, siR-93C@PAMAM, and HA-SeSe-COOH/siR-93C@PAMAM. (E) Western blotting analysis. (F) Semi-quantitative western blot analysis for the expression level of KRAS.

The results of above experiments confirmed that HA-SeSe-COOH/siR-93C@PAMAM did exhibit antitumor ability. Here, the inhibit effect in proliferation of A549 cells was further assessed, and flow cytometry was used to evaluate the cycle arrest and cell apoptosis of A549 cells (show in Figure 5(A–D)). In the tumor cell cycle arrest analysis (Figure 5(A,B)), compared with the control group (the proportion of G1-phase cells was 48.9%), the proportion of G1-phase cells in the HA-SeSe-COOH/siR-93C@PAMAM group was 72.2%, which proved that HA-SeSe-COOH/siR-93C@PAMAM could induce G1-phase cell cycle arrest in A549 cells. Meanwhile, the proportions of S-phase cells in siR-93C@Lipo2000, siR-93C@PAMAM, and HA-SeSe-COOH/siR-93C@PAMAM groups were 26.8%, 24.7%, and 20.4%, respectively.

In the cell apoptosis analysis results (Figure 5(C,D)), compared to the control group (1.13%), obvious early apoptosis could be achieved in HA-SeSe-COOH/siR-93C@PAMAM group (16.6%). Meanwhile, both siR-93C@PAMAM and HA-SeSe-COOH/siR-93C@PAMAM exhibited more powerful ability of inducing cell apoptosis (11.03% and 27.7%) than siR-93C@Lipo2000 (6.40%). Surprisingly, HA-SeSe-COOH/siR-93C@PAMAM exhibited stronger ability of inducing early cell apoptosis than siR-93C@PAMAM (2.70%). These results further confirmed that the HA-SeSe-COOH/siR-93C@PAMAM exhibited better siRNA transfection ability and can be applied for siRNA anti-tumor therapy.

### 3.10. Western blotting analysis

In order to further explore the molecular mechanism of A549 cell apoptosis induced by HA-SeSe-COOH/siR-93C@PAMAM complex, western blotting was performed to analyze the KRAS expression level in A549 cells after HA-SeSe-COOH/siR-93C@PAMAM transfection. Through Western blot analysis (in Figure 5(E,F)), the expression level of KRAS could be observed obviously decreased after the treatment with HA-SeSe-COOH/siR-93C@PAMAM complex, compared with the control group, the expression of KRAS decreased to 69.5% at 24 h and further decreased to 85.3% at 48 h, the knockdown of KRAS would contribute to the inhibition of A549 cells proliferation.

In the *in vitro* study, siR-93C was transferred into A549 cells by HA-SeSe-COOH/siR-93C@PAMAM, and the gene-silencing effect was evaluated by Western blotting assay. As shown in Figure 5(E,F), HA-SeSe-COOH/siR-93C@PAMAM treated group showed significant downregulation of the expression level of KRAS protein, which showed that the HA-SeSe-COOH/siR-93C@PAMAM could significantly silence the expression of the KRAS gene. In general, the HA-SeSe-COOH/siR-93C@PAMAM nanocomplex degraded quickly under redox environment to re-expose siR-93C@PAMAM, further PAMAM could facilitate the lysosomal escape of siR-93C via the proton sponge” effect. The siRNA-based gene silencing was activated in cancer cells, siR-93C induced about 90% knock-down of KRAS expression, significantly reduced cell viability, then inhibited proliferation of A549 cells, which was shown by the cycle arrest and apoptosis results (shown in Figure 5(A–C)).

### 3.11. *In vivo* distribution

**Figure 6.**
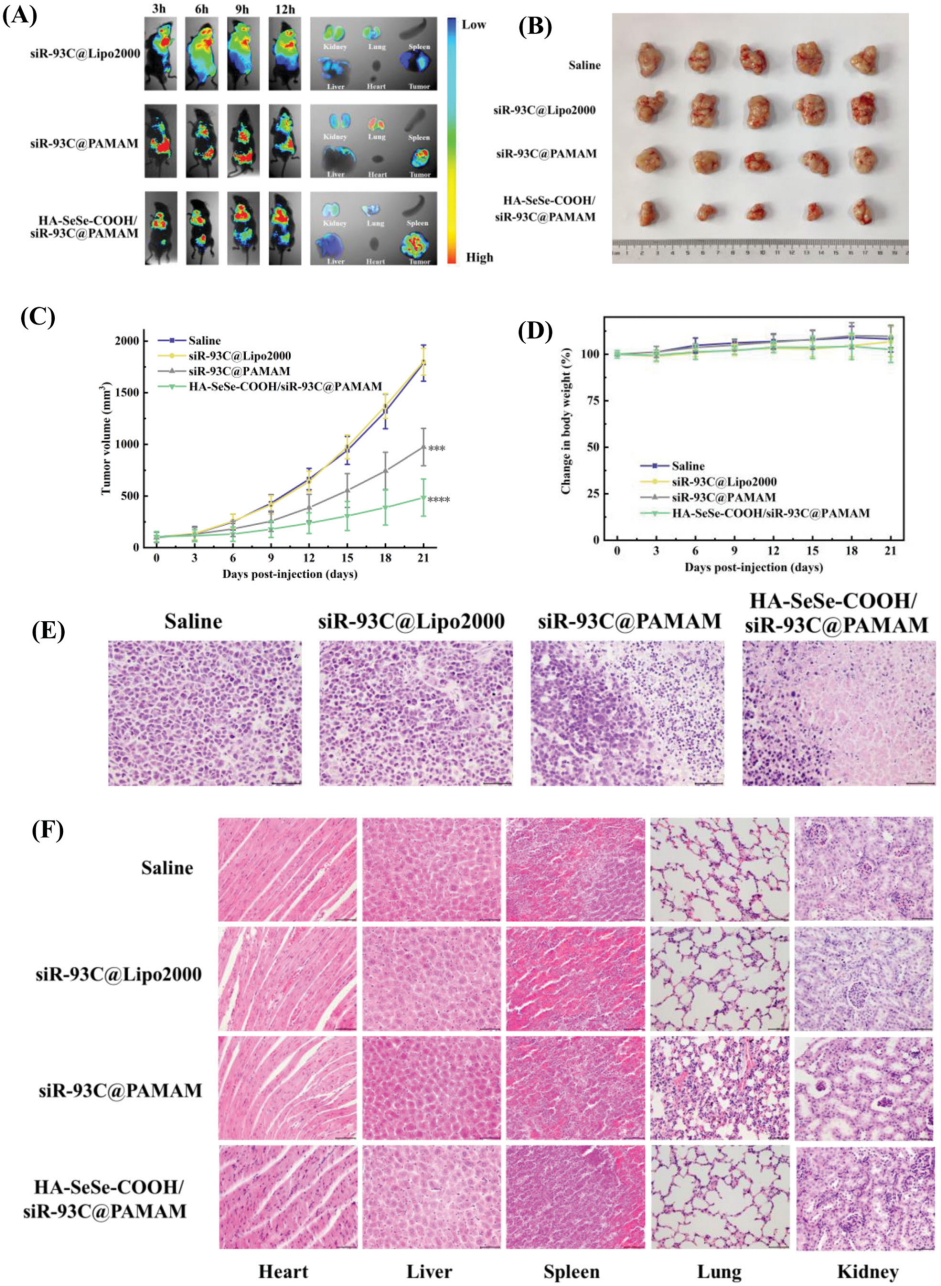
(A) *In vivo* distribution and imaging of siR-93C@Lipo2000, siR-93C@PAMAM and HA-SeSe-COOH/siR-93C@PAMAM in tumors-bearing nude mice and major organs. (B) Photograph of tumors dissected from each group at 21st day. (C) Tumor volume (mm^3^) of different groups. (D) Body weight changes of different treated groups. (E) Histological changes of tumor tissues after administration at 21st day (scale bar: 50 μm). (F) Histological H&E staining of major organs (heart, liver, spleen, lung and kidney) after treatment (scale bar: 50 μm).

To check the *in vivo* anti-tumor efficiency of HA-SeSe-COOH/siR-93C@PAMAM, different complexes with the same concentration of siR-93C-FAM was injected into tumor-bearing mice to investigate the *in vivo* distribution. As shown in Figure 6(A), though the siRNA fluorescence signal was observed in the tumor tissue at 3 h in siR-93C@Lipo2000 group, extensive fluorescence signal was also detected in the whole body (until 12 h), indicating that siR-93C@Lipo2000 didn’t exhibit specific tumor-targeting ability. In the siR-93C@PAMAM treated group, the tumor tissue exhibited fluorescence signal from 6 h till 9 h, and it significantly reduced at 12 h. It should be noted that the accumulation of siR-93C@PAMAM in the lungs and kidneys was significantly higher than other organs even up to 12 h after injection, indicated that siR-93C@PAMAM mainly passively accumulated in the tumor and distributed in the lung and kidney. On the other side, due to the positively charged property, siR-93C@PAMAM could be easily captured and rapidly eliminated by reticuloendothelial system (RES) and exhibited short circulation time after intravenous administration. As expected, tumor signal in HA-SeSe-COOH/siR-93C@PAMAM group was initially observed and accumulated at 3 h up to 12 h (fluorescence intensity was still extremely high after 12 h), which was due to the active targeting and binding ability of HA to CD44 (overexpressed on A549 cells). At the same time, the protective outer layer of HA-SeSe-COOH could allow complex a longer circulation time, consequently, increasing the accumulation within tumors (Ho et al., 2019).

### 3.12. *In vivo* anticancer activity and safety evaluation

Figure 6(B) shows the tumor volume of different treatment groups, negligible effects on tumor fast growth were observed in the treatment with siR-93C@Lipo2000 compare with saline group because of the limited bioavailability of siR-93C@Lipo2000. Excitingly, both siR-93C@PAMAM and HA-SeSe-COOH/siR-93C@PAMAM treated groups showed significant inhibition on tumor growth, with tumor volume considerably smaller than the saline group and siR-93C@Lipo2000 treated group. After 21 days treatment, the tumors of nude mice were harvested to evaluate the tumor growth inhibition effect (in Figure 6(C)). It was found that the HA-SeSe-COOH/siR-93C@PAMAM treated group showed the most remarkable suppression effect, as the tumor volume inhibitory rate reached to approximately 74.0%, showing that HA-SeSe-COOH/siR-93C@PAMAM could successfully inhibit the tumor growth. This result may be due to the efficient siRNA delivery and transfection. In addition, there was no observed weight loss of nude mice during the animal study period (in Figure 6(D)). Furthermore, according to H&E stained tumor slices images (Figure 6(E)), negligible level of necrotic cells was observed in saline and siR-93C@Lipo2000 treated mice. In contrast, obvious necrosis and inflammation was observed in siR-93C@PAMAM and HA-SeSe-COOH/siR-93C@PAMAM treated groups, this result was consistent with the inhibitory effect of A549 cells *in vitro* study.

To further evaluate the *in vivo* safety of HA-SeSe-COOH/siR-93C@PAMAM, the histological analysis of the main organs was performed and shown in Figure 6(F). No apparent tissue damage was observed in saline and siR-93C@Lipo2000 treated groups, however, collapse or disappearance of alveoli, irregular alveolar shape and thickening of the alveolar septa were observed in siR-93C@PAMAM treated group, indicating that the lungs of the siR-93C@PAMAM treated mice were severely damaged. Not only lung tissue but also certain level of kidney function was affected by siR-93C@PAMAM, some glomeruli were distorted or atrophied, tubular dilation and tubular epithelial cell necrosis and exfoliation were also observed. The major cause of the lung and kidney tissue damage was PAMAM. As mentioned above, PAMAM is cytotoxic due to its high density of positive charge which was attributed to the large number of terminal amino groups. *In vivo* distribution results have shown that siR-93C@PAMAM has strong lung accumulation and also certainly level of kidney accumulation. Taken together, it is not surprising that siR-93C@PAMAM would induce lung and kidney damage. As a contrast, insignificant inflammation and negligible necrotic cells could be observed in HA-SeSe-COOH/siR-93C@PAMAM treated group, indicating that the outer layer HA-SeSe-COOH did significantly improve the biosafety of PAMAM.

## 4. Conclusion

In summary, a redox-responsive siRNA targeted delivery vector, HA-SeSe-COOH/siR-93C@PAMAM, was constructed and employed as vector for KRAS siRNA transfection. Due to the superb internalization via clathrin-dependent pathway and excellent endosomal escape capability, HA-SeSe-COOH/siR-93C@PAMAM exhibited favorable transfection efficiency. Importantly, HA-SeSe-COOH/siR-93C@PAMAM could induce the cell apoptosis and the cell cycle arrest at G1 phase, leading to efficient anti-proliferative effect in A549 cells. Meanwhile, migration and invasion of A549 cells was also strongly inhibited. *In vivo* studies showed that HA-SeSe-COOH/siR-93C@PAMAM targeted and accumulated at the tumor site, effectively suppressed tumor growth and significantly reduced the toxic side effects of PAMAM. Thus, HA-SeSe-COOH/siR-93C@PAMAM could potentially be used as an effective carrier for siRNA delivery and transfection; it has great potential as gene carriers in tumor therapy.

## Supplementary Material

Supplemental Material

## Data Availability

All data and materials of this study can be obtained from the corresponding author upon reasonable request.

## Abbreviations

CLSM: Confocal laser scanning microscope
CPZ: Chlorpromazine
DAPI: 2-(4-amidinophenyl)-6-indolecarbamidine dihydrochloride
DMEM: Dulbecco’s modified Eagle medium
EIPA: 5-N-ethyl-N-isoproamiloride
FAM: Fluorescein amidite
FBS: Fetal bovine serum
FT-IR: Fourier transform infrared
GSH: Glutathione
HA: Hyaluronic acid
1H-NMR: Hydrogen nuclear magnetic resonance
H&E: Hematoxylin and eosin
Lipo2000: Lipofectamine™ 2000
MTT: 3-(4,5-dimethylthiazol2-yl)-2,5-diphenyltetrazolium bromide
EDC: N-(3-dimethylaminopropyl)-N′-ethylcarbodiimide hydrochloride
M-β-CD: Methyl-β-cyclodextrin
MFI: Median fluorescence intensity
NC: Negative control
N/P: Nitrogen to Phosphorus ratios
PAMAM: poly(amidoamine)
PBS: Phosphate-buffered saline
PDI: Polydispersity index
PI: Propidium iodide
RNAi: RNA interference
ROS: Reactive oxygen species
SeC: Selenocystamine
SD: Standard deviations
TEM: Transmission electron microscopy.
