# Supplementary material for: Hyaluronic acid-modified redox-sensitive hybrid nanocomplex loading with siRNA for non-small-cell lung carcinoma therapy
Source: Drug Deliv. 2022 Feb 14;29(1):574–87. doi: 10.1080/10717544.2022.2032874 (PMC8856077; doi:10.1080/10717544.2022.2032874)
Supplement: Supplemental Material [file IDRD_A_2032874_SM9557.docx]

Supporting information

**Hyaluronic acid-modified redox-sensitive hybrid nanocomplex loading with siRNA for non-small-cell lung carcinoma therapy**

Daoyuan Chen^a,1^, Peng Zhang^a,*,1^, Minghui Li^a,1^, Congcong Li^a^, Xiaoyan Lu^a^, Yiying Sun^b^, Kaoxiang Sun^a,*^

^a^ School of Pharmacy, Key Laboratory of Molecular Pharmacology and Drug Evaluation (Yantai University), Ministry of Education, Collaborative Innovation Center of Advanced Drug Delivery System and Biotech Drugs in Universities of Shandong, Yantai University, Yantai 264005 (P.R. China)

^b^ Shandong International Biotechnology Park Development Co.,Ltd, Yantai 264005 (P.R. China)

^1^ All authors contributed equally to this work.

* Corresponding authors: Dr. Peng Zhang and Prof. Dr. Kaoxiang Sun.

Mailing address: School of Pharmacy, Yantai University, 30 Qingquan Road, Yantai 264005, Shandong Province, China.

E-mail: peng.zhang@ytu.edu.cn (Dr. Peng Zhang), sunkx@ytu.edu.cn (Prof. Dr. Kaoxiang Sun).

Figure S1. Cellular localization of different siRNA nanocomplexes (siRNA and lysosomes were investigated).


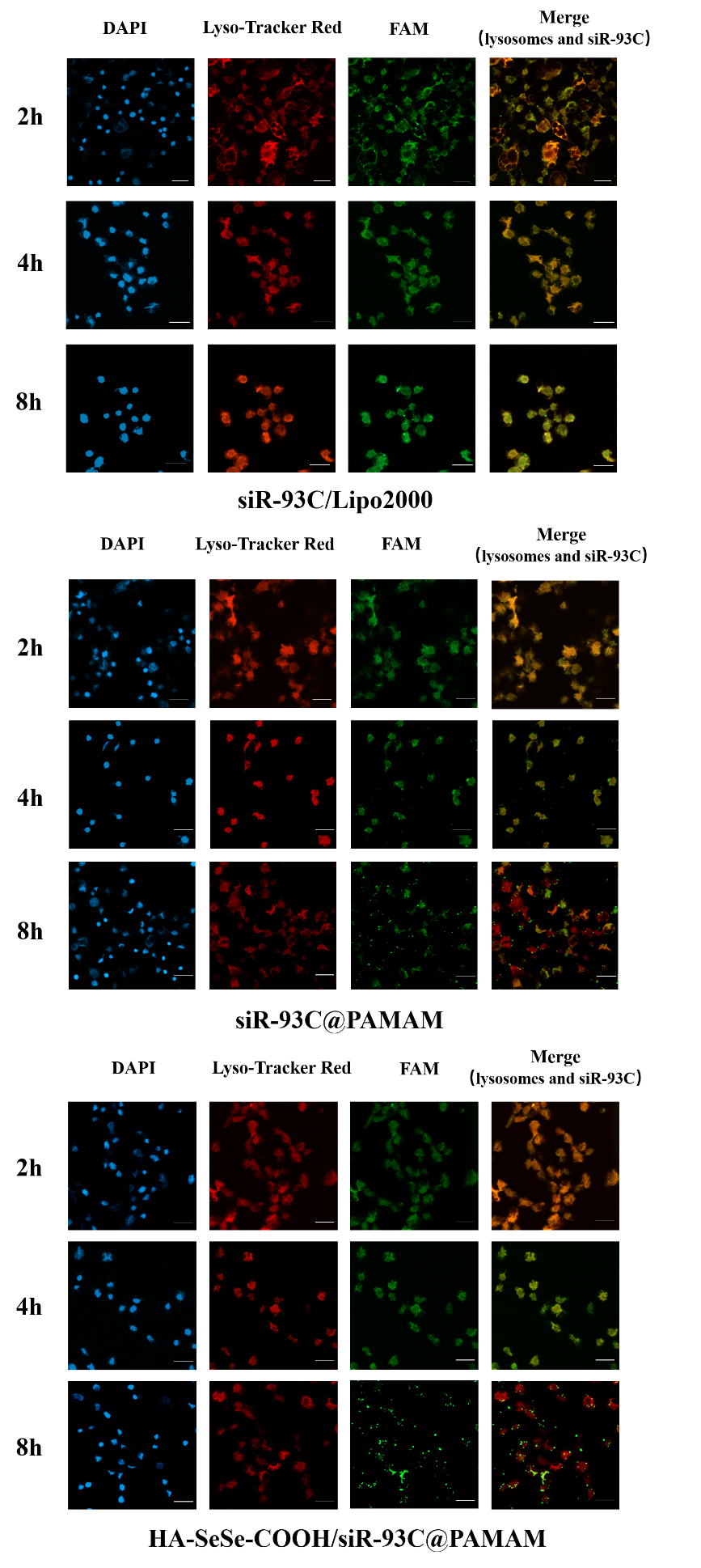


Figure S1. Cellular localization of different siRNA nanocomplexes. Nulcei, lysosomes and siRNA were labeled by DAPI (blue), Lyso-Tracker Red (red) and FAM (green), respectively. The scale bar is 100 μm.
